# Nurses’ Decisions to Press Charges Against Hypothetical Patients Who Exhibit Violent Behavior

**DOI:** 10.3390/nursrep16010035

**Published:** 2026-01-22

**Authors:** Darcy Copeland, Susan Tipton, Debra Culter, Mary Potter

**Affiliations:** 1 St. Anthony Hospital, CommonSpirit Health Mountain Region, Lakewood, CO 80228, USA; 2 School of Nursing, University of Northern Colorado, Greeley, CO 80639, USA; 3 Mercy Hospital, CommonSpirit Health Mountain Region, Durango, CO 81301, USA; 4 Avista Adventist Hospital, AdventHealth, Louisville, CO 80027, USA; 5 Parker Adventist Hospital, AdventHealth, Parker, CO 80138, USA

**Keywords:** workplace violence, patient violence, pressing charges, legal action, criminal charges, nurses, hospitals, ethics

## Abstract

**Background:** Patients are the most frequent perpetrators of physical violence against nurses. In the United States, most states have established laws designating assault against nurses a felony, or serious crime. It is unknown what reasons nurses have for pressing charges or not pressing charges against patients. **Purpose:** The purpose of this study was to examine nurses’ decisions regarding pressing charges when patients exhibit violent behavior. **Methods:** This study used a mixed-method, cross-sectional, descriptive design. Three unfolding case studies were presented in an electronic survey. Twelve versions of the survey were randomly assigned to participants. Each described an adolescent, adult, and geriatric patient. The narrative descriptions were identical, but the visual representations of the patients differed. **Results:** A total of 499 nurses from seven hospitals in the western United States responded. Most nurses indicated that they would not press charges against any of the hypothetical patients. An injury occurring and an assumption of intentionality contributed to nurses’ decisions to press charges. Participants were more likely to press charges against the adolescent and adult patients than the geriatric patient. The hypothetical adolescent and geriatric patients were more likely to have charges pressed against them if presented as female than if presented as male. The hypothetical adult patient was more likely to have charges pressed against them if presented as white than if presented as black. **Conclusions:** There is no consensus regarding when a nurse ought to pursue legal action against a patient who exhibits violent behavior. In addition to the presence of injury and the assumption of intentionality, it is possible that implicit bias may also play a role in these decisions. More investigation into this is needed.

## 1. Introduction

Physical violence is the most frequently reported type of workplace violence among nurses, and patients are the most frequent perpetrators [1,2,3]. Prevention strategies for patient violence include situational awareness, staff training in de-escalation [4], and altering the worksite to address wait times, crowding, alarms, and exit paths [5]. Fortunately, most incidents of workplace violence result in minor or no physical injury [3], though negative psychological or emotional consequences are not uncommon [6]. Known clinical risk factors for patient violence include altered mental status, intoxication/withdrawal, psychopathology, medication changes, delirium, pain, and brain injury [7,8,9]. When patients become violent, it is imperative that staff members respond to secure the environment and minimize harm to the patient, other patients, bystanders, visitors, and staff. Administration of emergency medications and initiation of physical restraints are sometimes necessary to re-establish a safe environment [10]. Nurses are encouraged to report instances of patient violence internally, although underreporting of violence against nurses is a long-standing problem [11].

In addition to internal workplace reporting, nurses have the option of reporting patient violence to law enforcement. Physical assault, regardless of the relationship between a victim and a perpetrator, is a crime. There are different legal categories of assaults, however. A simple assault, typically a misdemeanor offense, refers to an attack in which the perpetrator does not use a weapon and the victim does not sustain severe bodily injury. An aggravated assault, generally a felony offense, refers to an attack in which the intent is to inflict bodily harm and may involve a weapon [12]. In 2019 in the United States, among those arrested for aggravated assault, 76.5% were male and 23.5% were female [13]. In 2020, among juveniles arrested for simple assault, 62% were white, 34% were black, 2% were American Indian, and 1% were Asian. Among those arrested for aggravated assault, 57% were white, 38% were black, 3% were American Indian, and 1% were Asian. Among adults arrested for simple assault 65% were white, 30% were black, and 2% were American Indian or Asian. Among those arrested for aggravated assault, 62% were white, 33% were black, 3% were American Indian, and 2% were Asian [14].

In 2024 in the United States, 0.92% of people over age 12 were victims of simple assault and 0.31% were victims of aggravated assault [15]. Forty percent of the simple assaults and 69% of the aggravated assaults were reported to police. Males are more likely to be victims of assault, and this is reflected in reporting rates. Males reported simple assault to police at a rate of 7.2 per 1000 people and aggravated assault at a rate of 3.6 per 1000. Females reported simple assault to police at a rate of 4.5 per 1000 people and aggravated assault at a rate of 2.9 per 1000. Rates of simple assault victimizations reported to police were highest among 18- to 20-year-olds (11.2 per 1000 people), and rates of aggravated assault victimizations reported to police were highest among 21- to 24-year-olds (10.5 per 1000 people). Most simple assault victims who reported to police cited self-protection as the reason (31.9%), followed by civic duty (15%). Among the simple assault victims who did not report to police, the primary reasons were that they dealt with it another way (35.3%), the police could not/would not do anything to help (20.6%), or it was not important to do so (20.5%) [15].

In at least 32 US states, any physical assault against a nurse at work is considered a felony [16]. A rationale for promoting such laws is often deterrence; however, there is no evidence that states with these stipulations have experienced a decrease in violence against nurses because of the laws. It is also important for nurses to understand that in many cases, these laws include language that the perpetrator has knowingly or intentionally engaged in the assaultive behavior. If the patient who becomes violent has an altered mental status at the time of the assault, this can be difficult for criminal investigators or prosecutors to prove, and charges may be dropped. Nurses have reported feeling unsupported by police to pursue legal action because charges were unlikely to be pursued by prosecutors. In one study, only half of the complaints filed with police led to charges [17], so while the decision to press charges ought to lie with the nurse, perhaps police are not simply being unsupportive, but attempting to set realistic expectations regarding what might happen.

Nurses’ decisions to press charges against patients are made based on contextual factors. They are also inherently ethical decisions. Ethical aspects of the decision revolve around a nurse’s primary obligation to the patient and a balance between patient safety and nurse safety, the context in which a violent act occurred, including assessments of intentionality, the impact on the RN–patient relationship, and pressing charges because no other support is available as a victim. There is little known about hospital nurses’ decisions to press charges or seek legal action against patients who are violent. Most of the literature about pressing charges against patients comprises editorials, opinion pieces [18,19,20], incidental or unrelated findings in published studies [21], or is specific to violence perpetrated by patients in psychiatric settings [22,23,24]. There is very little research addressing hospital nurses’ pursuit of legal action as a primary topic under investigation. One study from Australia found that 27% of assaulted nurses filed police complaints and 28% of those charges resulted in findings of guilt. Reasons for not pursuing criminal charges included being discouraged to do so, lack of time, patient lack of mental capacity, feelings of guilt, and lack of harm [17].

The purpose of this study was to examine the effects of contextual factors, including a patient’s personal background (including age, gender, and race), medical diagnosis, and behavior, on nurses’ decisions to press charges when patients exhibit violent behavior. Pressing charges refers to the process of formally involving law enforcement or seeking legal action against another person. Secondary aims were to examine relationships between nurses’ exposure to workplace violence, expectations regarding workplace violence, self-assessment of risk tolerance, and decisions to press charges. Workplace violence, including patient violence, is conceptualized as a major occupational risk for nurses. Tolerance refers to “the action or practice of enduring or sustaining pain or hardship; the disposition to be patient with or indulgent to the opinions or practices of others; freedom from bigotry or undue severity in judging the conduct of others; forbearance; catholicity of spirit” [25]. The term “zero tolerance” is often used in reference to workplace violence involving nurses. There is, however, no empirical data indicating how tolerant nurses are to any occupational risks faced in their roles. For this reason, risk tolerance was included as a variable of interest in this study.

What is presented in this manuscript is analysis and findings related to nurses’ decision-making regarding pressing charges. Two other manuscripts addressing respondents’ perceptions of risk factors for, exposure to, and expectations of patient violence have been published [7,26].

## 2. Materials and Methods

### 2.1. Design

This study used a mixed-method, cross-sectional, descriptive design. The method chosen for this study was the presentation of unfolding case studies in an electronic survey. Unfolding case studies are widely used in nursing education to examine decision-making. They involve the presentation of information over time and require that the respondent evaluate and interpret new pieces of information and assess the need to make changes to a plan of care. A conceptual framework for developing cases for teaching purposes recommends attention to structure, process, and content and identifies attributes of relevance, realism, engagement, and challenge [27], which were used in the development of the cases in this study.

### 2.2. Sampling

Upon receipt of IRB approval, all registered nurses from seven hospitals within a healthcare system in the western United States were invited to participate via organizational email. Six hospitals are suburban, and one is in a rural community. The email was sent once per week for four weeks and included a link to an electronic survey. Completion of the anonymous survey implied consent to participate. When planning the study, the research team did not know how many nurses were employed at each facility. A Cochran formula was used to determine that a sample size of 385 was needed based on an unknown population of nurses employed across the participating hospitals, using a confidence interval of 95%, a margin of error of 5%, and a population proportion of 50%.

### 2.3. Instrument

The primary investigator has expertise in behavioral health and workplace violence and designed the case studies in collaboration with a geriatric and behavioral health clinical nurse specialist and a pediatric nurse ethicist. All three have experience participating on hospital workplace violence committees. The case studies all have elements of real incidents of patient violence and were designed to represent scenarios that hospital-based nurses may experience based on known risk factors for patient violence. The survey included three unfolding case studies describing an adolescent, an adult, and a geriatric patient to capture a range of patient ages that hospital-based nurses may work with. Participants were presented with personal, diagnostic, and clinical information in three stages. See Table 1 for case narratives. After each stage, participants were provided a text box and asked to identify what they thought the primary cause of the patient’s behavior was and whether they would press charges/seek legal action against the patient at this time, yes or no. There were 12 versions of the survey randomly assigned to participants, with four possible patient pictures associated with the three cases. Random assignment occurred as participants clicked the link to the electronic survey. They were asked what month they were born and were subsequently directed to a survey assigned to that month. Each of the case study narratives and corresponding questions were identical, the only difference was the picture of the hypothetical patient appearing next to the narrative. The adolescent patient was portrayed as a Latino, Latina, white female, or white male; the adult patient was portrayed as a black man, black woman, white woman, or white man; and the geriatric patient was portrayed as an Asian man, Asian woman, white woman, or white man. The images were obtained through Creative Commons to avoid copyright infringement. Attempts were made to locate images that depicted people in the same relative position (i.e., arms crossed or pointing), and presented with the same perspective (i.e., all waist up or all shoulders up). After the final stage, participants were asked, “If you sought legal action, why did you decide to do so when you did” and “If you did not seek legal action, why did you not?”

The survey utilized the Occupational Safety and Health Administration’s definition of workplace violence: “any act or threat of physical violence, harassment, intimidation, or other threatening disruptive behavior that occurs at the work site” [28]. Participants were asked about their frequency of exposure to any patient violence and to physical patient violence specifically. Three yes/no questions were also asked: Do you think it is possible to prevent patient violence in acute care facilities (hospitals) and get to zero instances of patient violence?, Do nurses in acute care facilities (hospitals) expect to be exposed to patient violence while at work?, and Should nurses in acute care facilities (hospitals) expect to be exposed to patient violence while at work?

The primary investigator also developed a self-report risk tolerance scale not specific to workplace violence for use in this study. Validated instruments used to assess individual risk tolerance associated with personal investment were examined and questions were altered with language about patient care. The questions were reviewed by a health and policy researcher with expertise in risk and decision-making. Questions asked on a five-point Likert scale, very low to very high, included: How would you rate your ability to tolerate stress associated with providing patient care, how would you rate your willingness to take risks, and what degree of personal risk are you willing to take when providing patient care? Questions asked on a four-point scale included: How easily do you adapt when things go wrong at work (very uneasily, somewhat uneasily, somewhat easily, very easily)? and When faced with a patient care decision, are you more concerned about patient safety or your safety (always patient safety, usually patient safety, usually my own safety, always my own safety)? Scores from these five questions could range from 5 to 23, with possible means ranging from 1 to 4.6. Mean scores of 2.3 or less were categorized as low risk tolerance and mean scores of 2.4 or higher were categorized as high risk tolerance. A pilot test of the survey with a group of nurses from one of the participating hospitals yielded a Cronbach alpha of 0.85.

### 2.4. Data Analysis

Descriptive statistics were used to summarize data. Chi squared tests were performed and odds ratios calculated to examine relationships between study variables. Data from incomplete surveys were retained and used in analysis. Analysis of the free-text responses followed Braun and Clark’s [29] steps for thematic analysis: familiarization with the data, generation of initial codes, searching for themes, reviewing themes, defining and naming themes, and writing. Textual responses were read in their entirety and then coded by all members of the research team individually. Codes were analyzed and organized into overarching themes independently before all team members came together to discuss and refine themes. Meeting notes were taken, and served as an audit trail for the group’s decision-making. Multiple team meetings occurred over a period of seven months to discuss each of the questions. These meetings were an important mechanism to reduce the potential for individual bias in reporting.

## 3. Results

### 3.1. Sample

A total of 499 nurses responded, yielding a response rate of 13%. Incomplete surveys were included in the analysis, so response rates might differ by question. Response rates to the 12 survey versions ranged from 22 to 38 (*M* = 29.8, SD = 4.4). Most respondents were staff nurses (251, 68%), followed by management (26, 7%), program coordinators and APRNs (7, 2% each), case managers (5, 1%), professional development and charge nurses (3, 1% each), nurse navigators (2, 0.5%), and house supervisors or “other” (1, 0.3% each). Respondents worked in the following departments: acute care (72, 19%), emergency department (53, 14%), intensive care (52, 14%), peri-anesthesia (44, 12%), labor and delivery (16, 4%), outpatient (15, 4%), other (13, 4%), interventional (10, 3%), inpatient rehabilitation (8, 3%), step down/progressive care (7, 2%), multiple units (7, 2%), palliative care (4, 1%), and float pool (3, 1%), with less than 1% in NICU, program-specific, community, case management, hospice, flights, quality, and wound care. Age, gender, race, and education sample demographics are presented in Table 2.

### 3.2. Survey Responses

Most respondents (433, 87%) reported exposure to workplace violence perpetrated by a patient and exposure to physical patient violence (351, 72%). Frequencies of exposure can be found in Table 3.

Most nurses (367, 85%) responded that they did not think it was possible to prevent patient violence in acute care facilities and get to zero instances of patient violence. While most respondents (350, 81%) thought nurses in acute care facilities *do expect* to be exposed to patient violence while at work, a minority (140, 32%) thought nurses in acute care facilities *should expect* to be exposed to patient violence while at work.

The mean risk tolerance score was 16 (SD = 2.5) and ranged from 8 to 23. Based on the cut point identified, 300 respondents (97%) were categorized as having high tolerance to work-related risk, and 9 (3%) as having low risk tolerance. Cronbach’s alpha for this sample was 0.65.

### 3.3. Decisions to Press Charges

The stages at which respondents indicated they would press charges against the hypothetical patients are presented in Table 4. If a respondent indicated they would press charges in a previous step, it was not included in subsequent steps. The results are therefore not cumulative. There were no significant associations between pressing charges against any patient at any stage and respondent demographics, frequency of exposure to violence, whether it was possible to prevent violence, whether nurses should expect to be exposed to violence, or risk tolerance.

**Adolescent Patient.** The primary reason respondents provided for Angel’s behavior at stage 1 was neurological disorder, at stage 2 it was IQ/mental deficit, and at stage 3 it was PTSD. Most respondents (191, 59%) indicated they would not press charges against Angel at any point. Among those who indicated they would press charges, the primary reason provided was that Angel had caused harm/injury. Among those who indicated they would not press charges, the primary reason was that Angel did not understand right from wrong. When Angel was presented as male, this lack of understanding was specifically associated with intellectual disability. See Table 5 for additional rationale. At the first stage, the odds of the hypothetical male patient having charges pressed against him were significantly lower than if the patient was portrayed as female (OR = 0.224, 95% CI [0.089, 0.565]). It is worth noting that in general, the language in the responses when Angel was presented as female had a harsher tone. They were referred to as “a brat,” “immature,” “acting out of defiance,” “acting out of boredom,” and “attempting to get attention.” This language was not noted when Angel was presented as male.

**Adult Patient.** When Rowan was presented as a white female, the primary reason respondents provided for the behavior at stage 1 was attitudinal: the patient was described as “a jerk,” “entitled,” “rude,” and “mean.” The primary reason provided for the other three patients was traumatic brain injury. At stage 2, the primary reason for the behavior was withdrawal and pain secondarily. The primary reason respondents provided for Rowan’s behavior at stage 3 was withdrawal. At this stage, when presented as male, the secondary reason was mental health disorder, and when presented as female, it was attitude and “personality.”

Most respondents (209, 60%) reported that they would not press charges at any point. The presumption of intentionality was one of the primary reasons participants provided for pressing charges against the adult patient. A lack of physical harm occurring was the primary reason participants gave for not pressing charges. See Table 6 for additional rationale. At stage 3, the odds of pressing charges against Rowan were significantly higher if the patient was white (OR = 1.77, 95% CI [1.047, 2.981]). With respect to the scenario presented, multiple participants commented that they “see this often” in their work. There were also comments indicating bias in the responses to this scenario, such as “I hate substance abusers.”

**Geriatric Patient.** The primary reason respondents provided for Jackie’s behavior at stage 1 differed by gender. The primary reason when Jackie was portrayed as female was fear related to the unfamiliar environment, and for males it was confusion related to Alzheimer’s. At stage 2, the primary reason was impaired communication, which exacerbated confusion and fear. At stage 3, the primary reason was infection/sepsis. When Jackie was portrayed as white, the secondary reason was hypoxia. When Jackie was portrayed as Asian, the secondary reason was confusion.

Most participants (327, 89%) indicated they would not press charges against Jackie at any point. The primary reason provided for pressing charges at any stage was that physical harm/injury occurred. Confusion, altered mental status, and lack of presumption of intentionality were primary reasons provided for not pressing charges. See Table 7 for additional rationale. The odds of pressing charges against Jackie were significantly lower if respondents reported that nurses in acute care settings do expect to be exposed to patient violence (OR = 0.438, 95% CI [0.213, 0.901]). There was a significant association between gender and decision to press charges at stage 1 (*X*^2^(1, N = 364) = 4, *p* = 0.045). All four of the hypothetical patients who participants indicated they would press charges against were women. Multiple respondents indicated that the scenario presented “happens all the time.”

## 4. Discussion

Given the multitude of previous studies indicating high rates of exposure to workplace violence, it is not surprising that most respondents reported exposure to physical patient violence. While no exposure is ideal, it is fortunate that this exposure to physical violence was relatively infrequent among this sample. It is also worth noting that most respondents did not believe it was possible to eliminate patient violence in hospitals. Many of the known risk factors for patient violence, as previously noted, are reasons patients may seek treatment in hospitals. Nurses are aware of these clinical and environmental risk factors for patient violence [7], which is potentially why most of these nurses do not think it is possible to eliminate it.

None of the case studies involved a patient with a weapon capable of causing severe bodily harm as described and whether any of the hypothetical patients intended to cause severe bodily harm was not explicitly stated. Approximately 40% of respondents reported that they would press charges against the adolescent and adult patients, which is consistent with simple assault reporting rates in the United States [15]. On the other hand, this sample’s decisions to press charges against female patients more often than male patients is not consistent with data from the general population, in which males represent a majority of those arrested [13].

The reasons participants provided for pressing charges against these hypothetical patients were not consistent with reasons reported by the general population either. In the general population, self-protection and civic duty are primary reasons for pressing charges [15]. These were not among the three primary reasons that participants in this study indicated they would press charges against any of the hypothetical patients. Among this sample, harm and the presumption of intentionality were among the most common reasons provided for seeking legal action.

It has been suggested, without evidence, that “nurses may not wish to press charges on patients with intellectual and developmental disabilities, patients who are cognitively impaired, or patients who are children” [18] (p. 3). The hypothetical geriatric patient was deemed cognitively impaired by most, if not all, participants in the present study, and only 11% reported that they would seek legal action. At the same time, however, 31% of respondents indicated that they would press charges against a child after discovering they had an intellectual disability. Based on this sample, nurses might in fact seek legal action against children and against patients with intellectual disabilities or cognitive impairments.

Given the discrepancy in decisions to seek legal action against these hypothetical patients, it is obvious that cognitive impairment may be one factor that nurses use when deciding whether to pursue legal action, but there are other factors that go into this decision. Harm and intentionality were two other important factors these respondents considered. Causing injury was a major factor in nurses’ hypothetical decisions to pursue legal action. As with cognitive impairment, however, it was not considered in isolation. The same proportion of respondents reported they would pursue charges against the adolescent and adult patients. Injury was the primary reason for the adolescent; however, injury was not a reason provided for that decision in reference to the adult patient. In the adult patient, history, presumed intentionality, and a need for accountability were reasons provided for pressing charges. The perception of intentionality and severity of harm have been noted to increase the likelihood of internally reporting violent events [30]. They are presumably relevant to decision-making regarding pursuing legal action as well. Harm and intentionality both appear to be positively related to decisions to press charges. If both are present, the decision to press charges is likely to increase. If both are absent, the decision to press charges is likely to decrease. Respondents indicated they would press charges when only one of these was present, however, making clear that the decision is multifaceted.

### 4.1. Limitations

The sample size of 499 respondents and a response rate of 13% is a limitation of this study. It is unknown how representative the sample is of the population of nurses at participating hospitals. Response bias is also a possibility. The topic of the study is sensitive, and it is possible that those who responded to the survey differ in unknown ways from those who opted to not participate. The survey was lengthy and required approximately 15 min to complete. It is also acknowledged that three questions per case study were repeated for participants. Responses were therefore not independent of one another. At the request of the organization, the survey was distributed in the month between two major holidays. Both factors potentially impacted participation rates. Additionally, while the survey was intentionally distributed to all RNs within the participating hospitals, nurses from different hospitals and different departments likely have varying levels of familiarity and comfort with initiating legal involvement. Examination of the effect of race and gender on decision-making was not direct, and some participants may not have noticed the picture displayed next to the narrative; however, one participant did make the following comment indicating that at least some participants noticed: “Stop making black women out to be angry and violent.”

### 4.2. Implications

There are multiple clinical and personal considerations in decision-making regarding seeking legal action against patients who have exhibited violent behavior. There are also several ethical considerations that ought to be acknowledged. Many of the known risk factors for patient violence are themselves reasons some patients require hospitalization. While there is no expectation that all violence can be prevented, healthcare professionals do possess specialized knowledge and skills to diagnose and treat some of the conditions associated with an increased risk of patient violence. What, if any, responsibility should a care team have if a patient is not accurately diagnosed or treated and then becomes violent? For example, where healthcare providers do not recognize that a patient has developed delirium or is withdrawing, and therefore does not intervene to correct the condition, and the patient becomes violent, who is culpable? Is it ethical to pursue charges against patients who become violent after being misdiagnosed and/or ineffectively treated? The provision of excellent clinical care is in staff and patients’ best interests. In some cases, it might alleviate the risk of violence. Optimal clinical treatment of all patients ought to be the highest priority and may be relevant to the occurrence of patient violence, and therefore any legal action that is taken as a result.

Most of the existing literature about seeking legal action against violent patients is related to psychiatric inpatients. Although related to psychiatry, concerns about a lack of systematic process in pursuing prosecution was raised decades ago, specifically that certain patients could be “singled out because of staff prejudice, hostility, or vindictiveness” [31] (p. 193). While most respondents in the present study indicated that they would not seek legal action against patients, it was more likely to happen if the hypothetical patients were female. The female patients in this study were described in disparaging terms not used to describe male patients. It is possible that there is an unconscious expectation that females ought to behave in certain ways, and when they do not, they are described in negative ways. This unconscious bias could also lead to nurses being more willing to tolerate violent behavior from males than females: violent behavior from males is perhaps expected and therefore tolerable, while from females it is less expected and therefore less tolerable.

Historically, rationales for punitively responding to crime in society include deterrence (punishing offenders deters others), retribution (offenders owe a debt to society), and incapacitation (offenders cannot offend while criminally detained) [32]. Although they were not the primary reasons participants in the present study provided for pursuing legal action, accountability and to a lesser extent preventing future violence were reasons some nurses cited in their decisions to press charges. Decision-making regarding pressing charges against violent patients is complex. There are clinical, ethical, situational, and personal factors that go into the decision. The decision is also known to cause anxiety, guilt, and stress [21,33], and is a personal one that must be left to the nurse to decide in the context of the situation [21,34].

At the same time, however, pressing charges ought to be done cautiously in the context of hospitalized patients. Pressing charges against a person can result in significant consequences for that person. If unconscious biases inform decisions about pursuing legal action, the repercussions seem significant. Some types of patients may be disproportionately represented among those against whom charges are brought. Policies that outline a formal process for nurses wishing to seek legal action against patients could increase consistency and identify potential bias. Such policies could be met with resistance, however, if perceived as a mechanism to prevent nurses from pursuing legal action if that is a decision they wish to make.

More than thirty years ago, the following questions were raised in reference to psychiatric staff members seeking legal actions against patients, and they remain relevant today and can be extended to hospitalized patients in general. What is the motive behind filing charges? What do you want to happen and why? Is retribution, although widely accepted in larger society, an acceptable motive for actions by treatment providers? Under what circumstances is it reasonable or unreasonable to insist that a patient be subject to the same societal consequences as non-patients? [35] (p. 43). These questions are not posed to dissuade staff members from pursuing legal action, but to sensitize them to some of the personal and ethical elements inherent in the decision.

## 5. Conclusions

Whether or not injury occurs and whether a violent act is interpreted as intentional or not contribute to nurses’ decisions to press charges against patients who are violent. Gender may also be an unconscious factor that contributes to the decision. Even in the presence of these contributing factors, however, the decision to pursue legal action is not universal, and most nurses would not press charges against patients. Deciding whether or not to press charges against a patient is a significant decision regardless of which decision is made. Multiple factors go into this decision, and no matter what decision an impacted nurse makes, it ought to be respected.

## Figures and Tables

**Table 1:** Case study narratives.

Adolescent	Stage 1: Angel, a 15-year-old, has been hospitalized on your unit for three weeks. Angel has functional neurological disorder and is unable to ambulate independently. Over the last week Angel has refused to participate with occupational/physical therapy. Additionally, rather than waiting for staff to assist in transferring to a commode Angel has begun rolling out of bed and flailing when staff attempt to help. It takes 4 staff members to safely return Angel to bed. During the time you are in Angel’s personal space providing care you have been spit at and slapped in the face.
	Stage 2: A social worker has informed you that based on IQ Angel has a mild mental disability. A wound on Angel’s forearm is not healing, and has become infected, because of continuous picking at it. When attempting to assess and care for this wound Angel spits, attempts to bite you, and slaps your arms, hands, and at your face.
	Stage 3: Angel also has a history of abuse and neglect and is diagnosed with posttraumatic stress disorder and reactive attachment disorder. During your most recent attempt to help Angel toilet, your arm was grabbed and wrenched behind your back injuring your shoulder. Multiple other staff members have experienced injuries resulting in needing to take time off work.
Adult	Stage 1: Rowan is a 36-year-old admitted to the hospital after being involved in an auto vs. pedestrian accident. After spending one day in the ICU following surgical repair of internal injuries and fractures Rowan is transferred to the medical/surgical unit where you work. Shortly after being transferred to your unit Rowan begins repeatedly calling for staff verbally (not using the call light) and demanding that you assist with getting food and returning to bed from the chair, change the sheets, fill the water bottle, and close the shades. During these encounters Rowan berates you, calls you names, accuses you of being a horrible nurse in a horrible hospital, makes threatening gestures, and threatens to sue you if you do not comply.
	Stage 2: The following day you find out that Rowan has a history of alcohol use disorder. Rowan continues to make demands of you and other staff members and is not participating in any requested care activities. You are called into the room roughly every hour and Rowan demands pain medication but refuses Protonix and Colace. You are providing regularly scheduled and PRN pain medication as often as is prescribed. When Rowan is informed that the maximum dose of prescribed pain medication has already been administered, Rowan throws a bottle of apple juice on the floor, screams expletives at you and threatens to hurt you if you do not administer additional pain medication.
	Stage 3: Rowan’s behavior continues to escalate. During each interaction with staff Rowan shouts, uses foul language, and threatens to harm staff. During one of your interactions Rowan threw a box of tissues across the room at you and on another occasion an empty water bottle; both items struck an extremity and did not cause you injury. Anything that can be removed from Rowan’s reach has been. This is Rowan’s third hospital admission this year. In reading the medical history you discover that Rowan has a history of behaving violently towards staff during each of these admissions.
Geriatric	Stage 1: Jackie is 88-years old and has resided at a residential facility for the past five years since becoming widowed. A grown son and daughter are unable to provide care in their own homes and do not have frequent contact with Jackie. Jackie has been diagnosed with Alzheimer’s disease and until recently was able to perform ADLs with supportive assistance. Two days ago, Jackie developed a fever and cough. Performing ADLs became progressively difficult, and Jackie began getting short of breath while ambulating around the facility. You are the nurse admitting Jackie to the hospital. While you are performing your initial assessment, Jackie begins to slap and pinch your arms and hands.
	Stage 2: Jackie is confused and continually questions what is happening. You reorient Jackie repeatedly and maintain your physical distance as much as possible. You have also learned that Jackie wears both hearing aids and glasses, neither of which were brought to the hospital. You complete your physical assessment and collect bloodwork and a portable chest x-ray. While doing so Jackie again slaps and pinches your arms and hands, this time more forcefully than the last, and attempts to hit your face and trunk.
	Stage 3: You have initiated oxygen therapy via mask and when Jackie removes the mask oxygen saturation drops into the low 80’s. Based on results of bloodwork and x-ray it is determined that Jackie has pneumonia and may be developing sepsis. Jackie begins screaming “I want to go to the bathroom” and is pulling at the gown and swatting at the air, flails in bed and attempts to remove the oxygen mask, IV, and blood pressure cuff. As you go to help, Jackie attempts to kick you, hits you hard in the back of the head and scratches your face drawing blood.

**Table 2:** Sample demographics.

		*n* (%)			*n* (%)
Age Group	20–29	46 (13%)	Long RN	<1 year	11 (3%)
	30–39	104 (28%)		1–5 years	81 (22%)
	40–49	66 (18%)		6–10 years	75 (20%)
	50–59	48 (13%)		11–20 years	77 (21%)
	60+	22 (6%)		>20 years	61 (17%)
	No Response	83 (23%)		No response	64 (17%)
Gender	Female	277 (75%)	Race and Ethnicity	Black/African American	2 (0.5%)
	Male	27 (7%)		Middle Eastern/North African	2 (0.5%)
	Nonbinary/Nonconforming	1 (0.3%)		Asian/Pacific Islander	7 (2%)
	No response	64 (17%)		Multiracial	8 (2%)
Education	ADN	37 (10%)		Native American/Alaskan Native	11 (3%)
	BSN	219 (59%)		White	249 (68%)
	MSN	43 (12%)		Hispanic/Latino	19 (5%)
	Doctoral	3 (0.8%)		No response	71 (19%)
	No response	67 (18%)			

**Table 3:** Frequency of exposure to patient violence.

	Multiple Times per Shift	Once a Shift	Once a Week	Once a Month	Couple of Times per Year	Once a Year	Every Couple of Years
Any violence	34 (9%)	29 (7%)	68 (17%)	85 (22%)	124 (31%)	24 (6%)	30 (8%)
Physical violence	12 (5%)	7 (2%)	31 (10%)	66 (22%)	102 (35%)	24 (8%)	54 (18%)

**Table 4:** Stages at which respondents would press charges against the hypothetical patient.

	Stage 1	Stage 2	Stage 3
Adolescent (n = 323)	31 (10%)	17 (5%)	84 (26%)
Adult (n = 348)	18 (5%)	38 (11%)	83 (24%)
Geriatric (n = 367)	4 (1%)	6 (2%)	30 (8%)

**Table 5:** Primary reasons for pressing charges or not, adolescent patient.

	Yes—Would Press Charges	No—Would Not Press Charges
White girl	Caused injury To prevent future violence To get her more services	Does not understand, is not responsible Nothing will be done, no point Would not help or change anything
Latina girl	Caused injury Knows right from wrong Continuous nature of violence	Does not understand, is not responsible Cannot control her behavior Would not help or change anything
White boy	Caused injury Was intentional, needs to be held accountable Required time off work	Does not understand, is not responsible Mental health history Nothing will be done, no point
Latino boy	Caused injury To get him more services Escalation of violence	Does not understand, is not responsible Age Cannot control his behavior

**Table 6:** Primary reasons for pressing charges or not, adult patient.

	Yes—Would Press Charges	No—Would Not Press Charges
White woman	Intentional History of violent behavior Will prevent future violence	No harm Has a disease and needs treatment No evidence crime was committed
Black woman	History of violent behavior Intentional Needs to learn lesson/needs consequences/accountability	No harm Has disease, clinical cause Not worth effort, no crime committed
White man	Physical abuse Intentional History of violent behavior	No harm Needs treatment Can just walk away or not engage
Black man	Physical abuse Needs to learn lesson/needs consequences/accountability Intentional	No harm Too difficult, not worth effort He can’t control his behavior

**Table 7:** Primary reasons for pressing charges or not, geriatric patient.

	Yes—Would Press Charges	No—Would Not Press Charges
White woman	Caused injury Not acceptable	Confused, does not understand what she’s doing Not intentional Part of disease process
Asian woman	Caused injury To document occurrence To protect others	Not intentional Confused, does not understand Nothing would change and would not hold up in court
White man	Caused injury Head and face involved To document occurrence	Altered mental status, does not understand Not intentional Part of disease process
Asian man	Caused injury	Altered mental status, does not understand Not intentional Part of disease process

## Data Availability

The data presented in this study are available on request from the corresponding author D.C. (Darcy Copeland) due to privacy restrictions. The data are not publicly available.

## Public Involvement Statement

There was no public involvement in any aspect of this research.

## Guidelines and Standards Statement

This manuscript was drafted against the STROBE guidelines for observational research. https://www.equator-network.org/ (accessed on 15 November 2025).

## Abbreviations

The following abbreviations are used in this manuscript.

APRN	advanced practice registered nurse
NICU	neonatal intensive care unit
ADN	associate degree in nursing
BSN	Bachelor of Science in nursing
MSN	Master of Science in nursing
IQ	intelligence quotient
PTSD	posttraumatic stress disorder
OR	odds ratio
CI	confidence interval
